# Mycelium-Doped Straw Biochars for Antibiotic Control

**DOI:** 10.3390/ijms252111387

**Published:** 2024-10-23

**Authors:** Bolun Zhang, Ruqi Li, Huiji Zhang, Ye Han, Yunzhe Jia, Siji Chen, Xiaoxiao Yu

**Affiliations:** 1 College of Life Sciences, Jilin Agricultural University, Changchun 130118, China; aa921526347@163.com (B.Z.); 14752267617@163.com (R.L.); zhanghuiji2001@163.com (H.Z.); hy2591203582@163.com (Y.H.); j18088671670@163.com (Y.J.); 2 Key Laboratory of Straw Comprehensive Utilization and Black Soil Conservation, Ministry of Education, Jilin Agricultural University, Changchun 130118, China

**Keywords:** mycelium doping, corn straw, biochar, antibiotics, adsorption

## Abstract

Straw, a predominant agricultural residue, represents a significant waste product. Harnessing its potential is of paramount importance both in terms of research and economic value. In this study, chemically pretreated corn straw was infused with distinct microbial fungal mycelium variants and subsequently transformed into a series of biochars through a process involving carbonization and activation. The findings revealed enhancements in the specific surface area and total pore volume of mycelium-doped straw biochars compared to the original corn straw biochar (BCS). Additionally, discernible disparities were observed in their physical and chemical attributes, encompassing functional groups, surface chemistry, and micro-morphology. Notably, in water-based antibiotic removal experiments focusing on tetracycline hydrochloride (TH) and chloramphenicol (CP), the mycelium-doped straw biochars outperformed BCS. Their maximum adsorption capacities for TH and CP surpassed those of alternative adsorbents, including other biochars. Impressively, even after five cycles, the biochar exhibited a removal rate exceeding 80%, attesting to its robust stability. This study successfully emphasized the efficacy of incorporating fungal mycelium to enhance the adsorption properties of straw-based biochar, introducing a new theoretical basis for the development of lignocellulosic materials.

## 1. Introduction

Straw, as a significant by-product of staple crops, harbors undeniable potential as a biomass resource [1]. China, being a traditional agricultural powerhouse, boasts an annual straw output of 900 million tons. According to data released by the National Bureau of Statistics, corn straw (CS) constitutes nearly one-third of the total straw output, thus warranting unparalleled significance in delving into comprehensive CS treatment technologies, vigorously fostering new preparatory techniques, and cultivating high-value products derived from straw. Throughout the past few decades, our team has been dedicated to exploring the multifaceted utility of straw, aiming to generate advanced, value-added products such as gluconic acid [2], sophorolipids [3], and D-lactic acid [4] from CS. Furthermore, our focus extends to crafting biochars of exceptional performance using CS as a raw material, aimed at addressing issues of energy storage and environmental pollution (synthetic dyes, heavy metals, and antibiotic residues); straw biochars have the advantages that most adsorbents cannot match, such as huge specific surface area, rich functional groups, etc. [5,6]. Nonetheless, our ongoing mission remains the relentless exploration of new technologies to fully unlock the potential of straw.

The conversion of straw into biochar represents a more efficient approach to augment its added value. However, the performance of the resultant straw biochar stands as a pivotal determinant influencing its practical applicability and economic potential. To enhance the physicochemical properties of biochar derived from straw, numerous scholars have employed diverse pretreatment methods. Whether employing chemical [7], physical [8], or biological approaches [9], the ultimate objective remains consistent: the disruption of the original lignocellulosic structure of straw to render it more amenable to processing. While the efficacy of pretreatment is indisputable, its impact extends beyond mere structural alteration. Drawing upon our previous investigations, we have discerned that effective pretreatment techniques can selectively eliminate specific lignocellulosic components [10,11]. For instance, treatments such as potassium hydroxide for lignin removal, hydrochloric acid for hemicellulose hydrolysis, and solutions comprising urea and alkali for cellulose dissolution have been identified as proficient methods [4,11], revealing a nuanced capability to target and expose distinct components within the straw matrix. However, this improvement has its upper limit; for example, no matter what preparation method is used to make a biochar adsorbent from the pretreated straw precursor, its adsorption performance cannot be further improved (whether by increasing the proportion of the activator or increasing the activation temperature) [10,11]. Therefore, further processing is required to further improve its performance to maximize the value of the biomass.

Modification is a viable approach for significantly further enhancing the efficacy of biochar [12,13,14]. The common methods of straw biochar modification include the addition of charcoal, functional modification, and doping modification. Acids and bases are commonly used to remove minerals and silicon impurities from biochar. To make biochar functional, it is common to use sources that are oxygen-rich (such as hydrogen peroxide) or rich in amino drugs (polyamines). Generally, doping modification does not require specific reagents, and the wide range of sources of doping materials is almost unlimited, which attracts more scholars. Mycelium, 3D network structures formed by fungal spores via meristem, are rich in monomer compounds (e.g., anthraquinone, sterols, and aromatic hydrocarbons) and functional groups [15,16,17]. Given the problem of cost and simple operations, it seems to be a better choice to dope biochar with microbial fungal mycelium compared with most inorganic doping and organic synthesis processes. But little studies have been reported, which encourages us to explore this interesting area.

In this study, three kinds of pretreated CSs were used as raw materials, and then mycelium-doped pretreated CSs were obtained by a gentle fermentation process, and finally, mycelium-doped modified straw biochars with further improved adsorption properties were prepared via carbonation activation. Scanning electron microscopy (SEM), N_2_ adsorption–desorption, Fourier transform infrared spectroscopy (FT-IR), X-ray diffraction (XRD), Raman spectroscopy, Zeta potential, and X-ray photoelectron spectroscopy (XPS) were used to study the physicochemical properties of straw biochar modified by different forms of mycelium doping. Tetracycline hydrochloride (TH) and chloramphenicol (CP) were selected as adsorbent models, and the effects of contact time, initial concentration, temperature, and pH value were investigated to study the adsorption properties of mycelium-doped biochars. This work aimed not only to develop a series of straw-based biochars with excellent adsorption properties that can effectively remove antibiotics from water. More importantly, it provides valuable experimental evidence and theoretical reference to verify that fungal mycelium doping can further improve the performance of biochar, and provides a new strategy for the further development of agricultural by-product straw lignocellulosic biochar.

## 2. Results and Discussion

### 2.1. Preparation of Mycelium-Doped Straw Biochars

**Figure 1:**
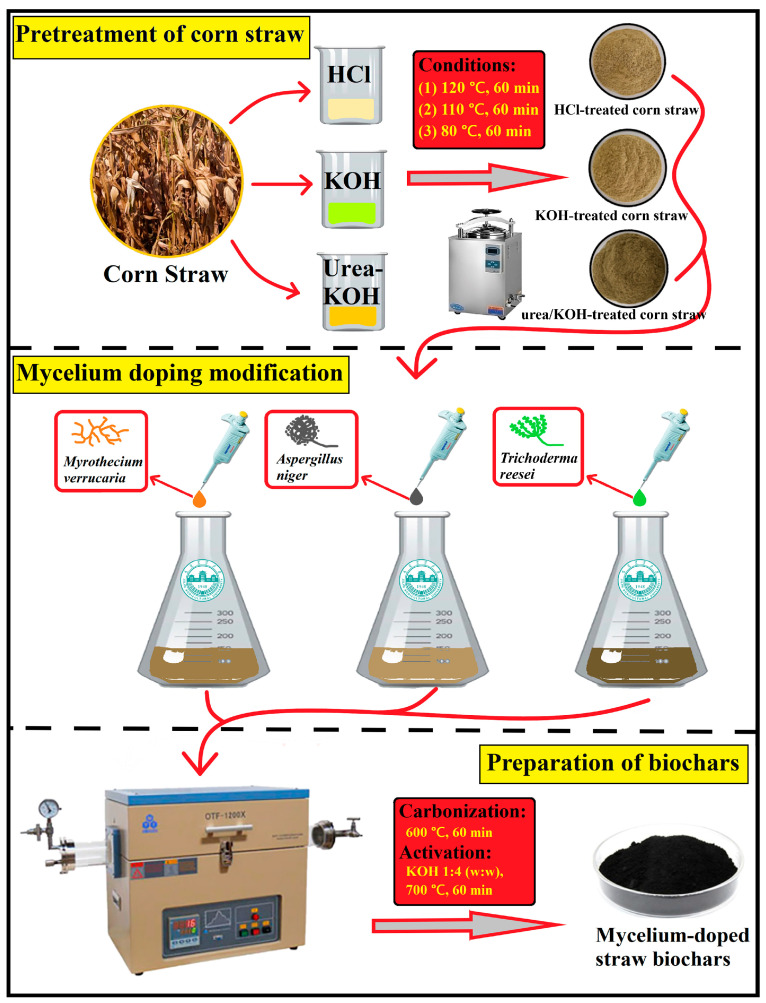
Scheme of the preparation of mycelium-doped straw biochars.

The preparation process of mycelium-doped straw biochar is illustrated in Figure 1. Generally, the purpose of chemically pretreating straw lignocellulosic materials is to disrupt the natural dense structure, enhancing its efficiency for utilization. The results (Table S1, Supplementary Materials) indicate significant changes in the lignocellulosic components of the treated straw compared to the original, affirming the success of the chemical treatment process. This success lays a solid foundation for the subsequent doping modification with fungal mycelium. The main production enzymes of *Myrothecium verrucaria* (*M. verrucaria*) are laccase, lignin peroxidase, and manganese peroxidase, which could effectively degrade lignin. *Aspergillus niger* (*A. niger*) exhibits high xylanase activity, a crucial enzyme for hemicellulose degradation. The cellulase produced by *Trichoderma reesei* (*T. reesei*) is an effective agent for degrading crystalline cellulose [18]. To minimize excessive damage to the straw’s structure and composition during microbial decomposition and complete the mycelium doping modification in a controlled and gentle manner, we adopted the strategy of reducing effective carbon sources. Microorganisms were introduced into the medium with the least content of suitable effective carbon sources (cellulose, hemicellulose, and lignin) for their growth. Consequently, various microbial fungi, including *A. niger*, *T. reesei*, and *M. verrucaria*, were added to prepare the pretreated straw (CS-H, CS-U, and CS-K) medium. In this process, saprophytic fungi utilized the straw as a carbon source, secreting enzymes to degrade it. Simultaneously, the mycelium accomplished doping modification of the pretreated CS throughout the solid fermentation process by inserting, winding, and binding. Finally, the mycelium-doped straw biomass (CS-H-A, CS-U-T, CS-K-M) underwent cleaning and drying, followed by the preparation of biochar using the carbonation activation method.

### 2.2. Characterization

**Figure 2:**
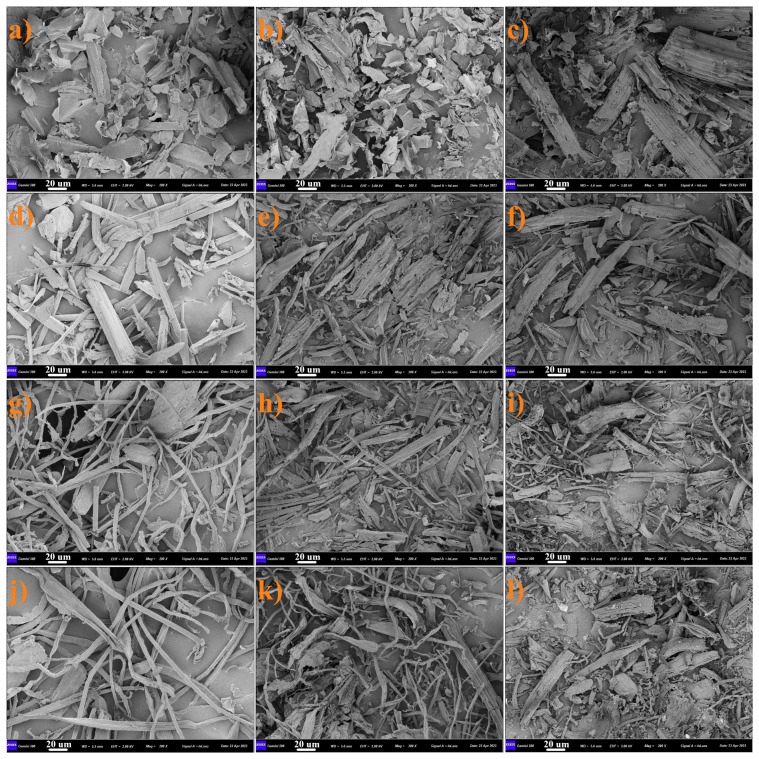
The scanning electron microscopy (SEM) imagines of (**a**) corn straw, (**b**) carbonized corn straw, (**c**) corn straw biochar, (**d**) urea/KOH-*T. reesei*-treated corn straw, (**e**) carbonized urea/KOH-*T. reesei*-treated corn straw, (**f**) urea/KOH-*T. reesei*-treated corn straw biochar, (**g**) KOH-*M. verrucaria*-treated corn straw, (**h**) carbonized KOH-*M. verrucaria*-treated corn straw, (**i**) KOH-*M. verrucaria*-treated corn straw biochar, (**j**) HCl-*A. niger*-treated corn straw, (**k**) carbonized HCl-*A. niger*-treated corn straw, and (**l**) HCl-*A. niger*-treated corn straw biochar.

It can be seen from the SEM image that after the smooth surface of CS (Figure 2a) is carbonized at high temperature, the surface of CCS (Figure 2b) shows obvious dehydration phenomenon and begins to produce folds and fragmentation. With further activation, the more violent reaction resulted in a large amount of etched debris on the surface of the BCS (Figure 2c). The mycelium-doped straws CS-U-T (Figure 2d), CS-K-M (Figure 2g), and CS-H-A (Figure 2j) retained the woody fiber structure of the straw but also obviously had some filamentous structures, which were mycelium from *A. niger*, *T. reesei*, and *M. verrucaria* with a diameter of 2–5 µm. After carbonization, these mycelia (Figure 2e,h,k) began to become thinner and began to partially fracture, indicating that they had a significant dehydration phenomenon in the process of pyrolysis at high temperature. More breakage of the mycelium occurred in the activated sample, and it was clear that most of the mycelium became shorter fragmented rods. In spite of this, a large number of mycelia still existed in the biochar after carbonization and activation, indicating that the modification of straw by mycelium through the microbial solid fermentation process was still successful.

**Figure 3:**
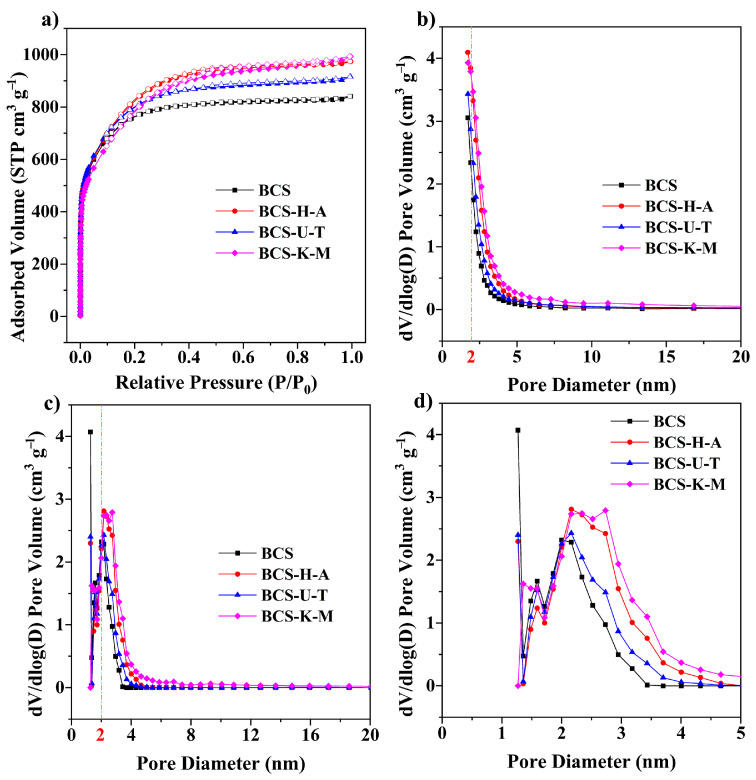
(**a**) N_2_ adsorption–desorption isotherms of biochars. The pore size distribution curves of biochars based on (**b**) Barrett–Joyner–Halenda and (**c**) non-local density functional theory range from 0 to 20 nm. (**d**) The pore size distribution curves of biochars based on non-local density functional theory range from 0 to 5 nm.

N_2_ adsorption–desorption isotherm tests were performed on the samples to evaluate the pore structure characteristics of the biochar (Figure 3 and Table S2). The Brunauer–Emmett–Teller (BET) theory was used to calculate the surface area. The non-local density functional theory (NLDFT) and the Barrett–Joyner–Halenda (BJH) model were used to analyze the pore size distribution of samples. All the samples showed I and IV isotherm models, indicating that the prepared biochars were porous carbons with microporous and mesoporous structures [19,20], which is consistent with the results of Figure 3b–d. The BET specific surface area (S_BET_) of BCS is 2429 m^2^ g^−1^ and the total pore volume is 1.30 cm^3^ g^−1^. At the same time, the S_BET_s of BCS-U-T, BCS-K-M, and BCS-H-A are 2604, 2790, and 2715 m^2^ g^−1^, respectively, and the corresponding total pore volumes (V_total_) are 1.42, 1.51, and 1.54 cm^3^ g^−1^. The S_BET_ of BCS is lower than that of mycelium-modified straw biochar (BCS-U-T, BCS-K-M, and BCS-H-A), which may be due to two reasons. On the one hand, the decomposition of pretreated straw by microorganisms in the process of solid fermentation makes the straw structure broken and fluffy, and part of the macroporous structure is generated, which greatly increases the contact area between the activator and carbonizing precursor and promotes the activation efficiency of the activator; this, in turn, creates a more porous structure. In addition, mycelium, as a micron-scale filamentous substance, also becomes thinner during carbonization and activation, resulting in fracture, etching, and a porous structure (Figure 2d–1), which on the other hand improves the activation efficiency and further increases the specific surface area of the biochar. Mycelium doping modified straw can increase the S_BET_ of straw-based biochars by 7–15% and the Vtotal by 9–18%, showing that the mycelium doping modification is an effective way to further improve the physicochemical properties (especially the pore structure) of straw biochar.

**Figure 4:**
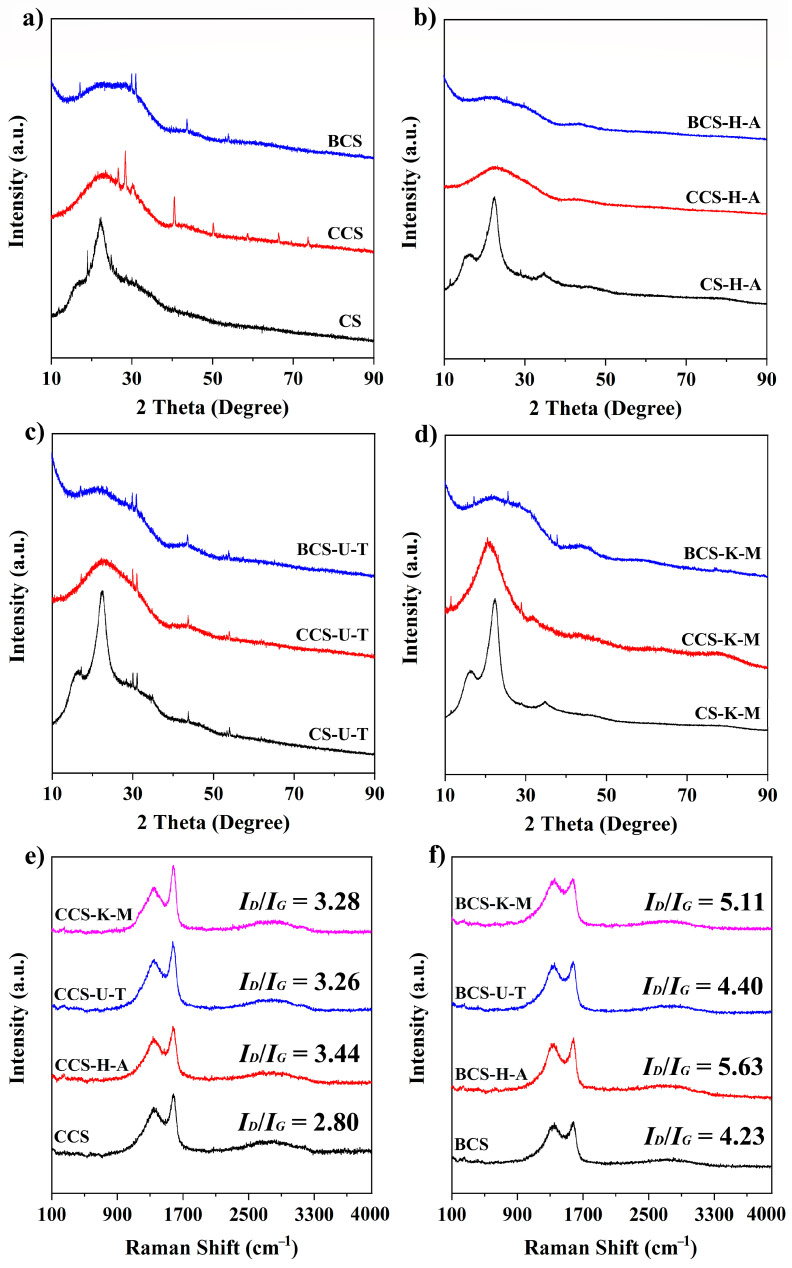
X-ray diffraction (XRD) tests of (**a**) CS, CCS, and BCS; (**b**) CS-H-A, CCS-H-A, and BCS-H-A; (**c**) CS-U-T, CCS-U-T, and BCS-U-T; (**d**) CS-K-M, CCS-K-M, and BCS-K-M. Raman spectra of (**e**) CCS, CCS-H-A, CCS-U-T, and CCS-K-M; (**f**) BCS, BCS-H-A, BCS-U-T, and BCS-K-M.

The crystallinity and the presence of defects in the carbon of biochar were detected by XRD tests and Raman spectra (Figure 4). The peaks at 17° and 23° represent the lignocellulosic structure of the straw [6], which was consistent in both CS and mycelium-modified CSs. There are some irregular peaks that become sharper and more pronounced after carbonization, which may be due to the incorporation of inorganic salts ((NH_4_)_2_SO_4_, KH_2_PO_4_, MgSO_4_, CaCl_2_, NaCl, FeSO_4_, MnSO_4_, and ZnCl_2_) in the preparation of the medium during solid fermentation [21]. These peaks become less intense after activation; the main reasons for this may be two-fold: one is the alkaline activator used in high-temperature etching of carbon precursors, and the other is the dilute acid used (0.1 M HCl) and the large amount of deionized water involved in washing after the activation is completed. The amorphous structure of the biochar is indicated by the presence of (002) and (100) graphite structure planes at 10 to 30° and 40 to 50°, respectively. Raman measurements revealed two typical peaks: a D-band with amorphous carbon at 1361 ± 2 cm^−1^ and a G-band with graphite carbon at 1583 ± 4 cm^−1^ [22]. An essential index to gauge the degree of carbon disorder and defects is the intensity ratio between the D-band and G-band (I_D_/I_G_). The I_D_/I_G_ values of CCS, CCS-H-A, CCS-U-T, and CCS-K-M were 2.80, 3.44, 3.26 and 3.28; these values were lower than that of activated biochars BCS, BCS-H-A, BCS-U-T, and BCS-K-M (4.23, 5.63, 4.40, and 5.11), indicating that the activation process produced more amorphous carbon structures. In addition, there is an interesting rule: whether it is the carbonized sample or activated sample, the I_D_/I_G_ ratio of the mycelium-doped straw is higher than that of the original straw. These results once again support our hypothesis that chemical pretreatment and microbial decomposition processes destroy the straw’s dense and organized natural structure, which means that the doping modification of the mycelium is effective. However, there is no doubt that these results show that the biochars contain both graphite and amorphous carbon structures; in other words, biochars derived from straw or straw treated with mycelium exhibited a characteristic local-order structure commonly observed in carbon compounds.

The functional groups of the biochars were studied using FT-IR, and the results are displayed in Figure S1. The wide band corresponding to the -OH stretching vibration of hydroxyl functional groups in the region of 3450–3410 cm^−1^ is still present, showing that the produced biochars contain residual oxygen-containing functional groups [5]. Carbonized and activated samples have weaker -CH bonds (2910–2930 cm^−1^, symmetric and asymmetric tensile vibration peaks of -CH groups) than CS, CS-H-A, CS-U-T, and CS-K-M, indicating that a significant quantity of hydrogen is lost during carbonization and activation [16]. The aromatic ring’s C=C stretch is highlighted by the peak at 1610–1620 cm^−1^ [23]. This can be explained by the fact that the C-H bond may have broken down following high-temperature processing into the more stable aromatic C=C bond. This could be because the carbonyl group is produced when the C=O group joins with the aromatic ring during the aromatization process [24]. Furthermore, overlapping bands in the 1000–1400 cm^−1^ range correspond to C-O and C-N heterocycle stretching vibrations. C-C skeletal vibration, bending vibration of oxygen-containing groups (-CH, O-H), and tensile vibration of some single bonds (C-O, C-N) yield the typical peaks below 1100 cm^−1^ [25]. The presence of these large functional groups may improve the performance of biochars as adsorbents. XPS testing was used to detect the chemical valence of surface functional groups of biochar (Figure S2). The C1s spectra can be divided into three characteristic peaks corresponding to C-C, C-O, and C=O [26]. The O1s spectra consist of five characteristic peaks for quinones or metal oxides, C=O, C-O, -OH, and O=C-O [27,28]. These rich carbon- and oxygen-containing functional groups may improve biochar adsorption.

When the overall charge density of the outer coordination complex equals zero, the variable surface charge and the constant charge dictate the point at which the charge becomes zero. This point is also known as the pH (pH_pzc_), inner coordination complex charge density, constant charge density, and proton interaction charge density [29,30]. When pH is less than pH_pzc_, the sample’s surface is positively charged, and vice versa. BCS, BCS-H-A, BCS-U-T, and BCS-K-M surface charges were determined by zeta potential analysis (Figure S3). In general, with the increase in the pH value of the solution, the surface of biochars carries more and more negative charges, and the potential also becomes lower; furthermore, the pH_pzc_ values of BCS, BCS-H-A, BCS-U-T, and BCS-K-M are 4.97, 4.73, 3.34, and 4.32, respectively. To some extent, the pH_pzc_ of all the straw biochars modified by mycelium doping (BCS-H-A, BCS-U-T, and BCS-K-M) are slightly lower than that of straw biochar (BCS). We speculate that this might be caused by the existence of mycelium or the difference in the lignocellulose composition of the straw caused by pretreatment. In a follow-up study, we will further explore this interesting phenomenon. There is no doubt that biochars have great potential for treating cationic pollutants at higher pH values.

### 2.3. Adsorption Experiments in Single System

**Figure 5:**
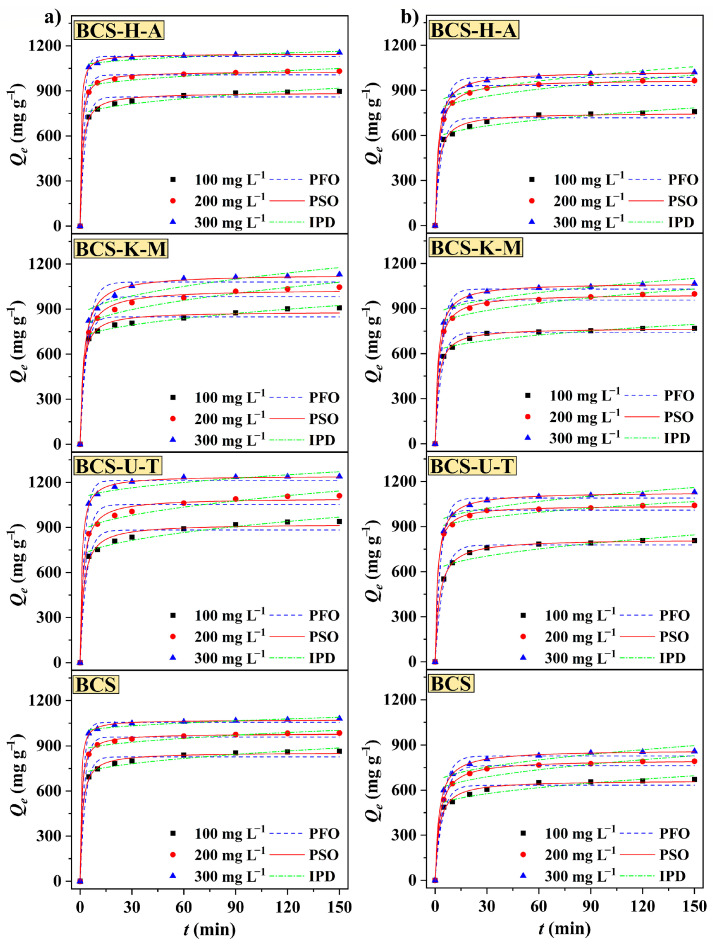
The adsorption plots of BCS, BCS-H-A, BCS-U-T, and BCS-K-M fitted with adsorption kinetic models for (**a**) TH and (**b**) CP at 303 K (dose of biochars: 10 mg; *V*: 200 mL; temperature: 303 K; pH: 7.0; shaker speed: 150 RPM).

Adsorption kinetics summarize the physical and chemical reactions that occur during adsorption between the adsorbent and the adsorbate [31]. Details of the adsorption experimental methodology and fitting equation modeling are provided in Supplementary Material S3. In general, the adsorption capabilities of BCS, BCS-U-T, BCS-K-M, and BCS-H-A grew dramatically in the first 5 min and gradually achieved equilibrium at 60 min (Figure 5). Extending the contact duration to 120 or 150 min was not effective in further increasing the adsorption capacities. To investigate the control mechanism of chemical processes during adsorption, this work employed three widely used adsorption kinetic models: Lagergren’s PFO model, Ho–McKay’s PSO model, and Weber–Morris’s IPD model. The experimental data (Table S3) were analyzed using these models. The PFO model was utilized to investigate the adsorption processes, and the *R*^2^ values for TH, CP, BCS, BCS-U-T, BCS-K-M, and BCS-H-A were in the ranges of 0.9802–0.9869, 0.9676–0.9770, 0.9747–0.9774, and 0.9856–0.9873. Meanwhile, the low *Q_e.cat_* indicates that the PFO model is not suitable for describing the adsorption process. When fitting the adsorption process with the IPD model, BCS, BCS-U-T, BCS-K-M, and BCS-H-A have correlation coefficients of 0.7952–0.8834, 0.7652–0.9331, 0.8164–0.9556, and 0.8001–0.8652 for TH. However, CP’s correlation values ranged from 0.7407 to 0.8794, from 0.7416 to 0.7626, from 0.7467 to 0.8061, and from 0.7406 to 0.8794, indicating that IPD did not dominate adsorption. As for the PSO model, the *R*^2^ values of biochars were in the range of 0.9971–0.9996, 0.9913–0.9993, 0.9918–0.9970, and 0.9973–0.9996 for TH and 0.9929–0.9998, 0.9992–0.9999, 0.9988–0.9998, and 0.9939–0.9998 for CP. In addition, the theoretical adsorption capacities *Q_e.cat_* are also slightly greater than the actual *Q_e_*, hence suggesting the suitability of Ho–McKay’s model in the adsorption procedure. We can deduce that the adsorption mechanisms of biochars for TH and CP may involve chemical reactions, as the adsorption behaviors between adsorbents and adsorbates, such as transfer, exchange, or sharing, can lead to the formation of chemisorption bonds, which may govern the rate of adsorption [32,33]. Additionally, it is worth noting that a higher concentration of the solution has shown some effectiveness in enhancing the adsorption capacity to a certain extent. We can also achieve a short conclusion that the adsorption capacities of mycelium-doped modified straw biochar (BCS-U-T, BCS-K-M, and BCS-H-A) are slightly greater than that of BCS, which once again proves the effectiveness of this modification method for improving the properties of biochar materials.

**Figure 6:**
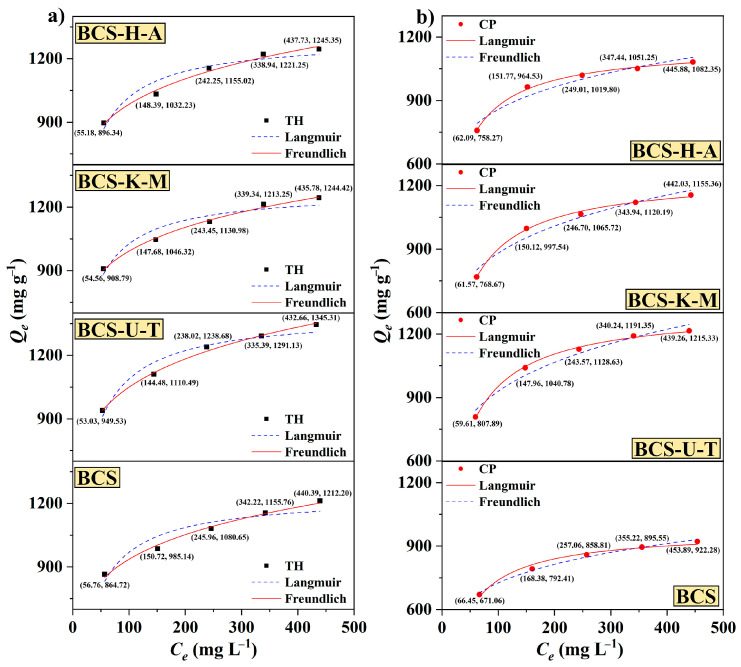
The adsorption isotherms of BCS, BCS-U-T, BCS-K-M, and BCS-H-A to (**a**) TH and (**b**) CP at 303 K (dose of biochars: 10 mg; *V*: 100 mL; temperature: 303 K; pH: 7.0; shaker speed: 150 RPM).

The adsorption isotherm characterizes the relationship between concentration and adsorption capacity. In this study, we investigate the interactions between biochars and the adsorbates (TH and CP) at a temperature of 303 K (Figure 6). Various initial concentrations of the solution are examined to understand the behaviors during the processes. It can be clearly seen that no matter whether biochar is modified by mycelia or not, whether the adsorbent is TH or CP, their adsorption capacity increased with an increase in solution concentration within a specific range. In order to further explore the factor which affected the adsorption, the data were fitted using the Langmuir isotherm, which is commonly used to describe the homogeneous molecular adsorption process [34], and the Freundlich isotherm, which is used to describe the heterogeneous multi-layer adsorption process [35] (Table S4, Supplementary Materials). The *R*^2^ for BCS, BCS-U-T, BCS-K-M, and BCS-H-A was found to be 0.8942, 0.9490, 0.9223, and 0.9293, respectively, when the Langmuir model was applied. These values were all lower than the *R*^2^ obtained using the Freundlich model, which was 0.9908 for BCS, 0.9950 for BCS-U-T, 0.9951 for BCS-K-M, and 0.9982 for BCS-H-A. This suggests that the adsorption processes of biochars for TH were not characterized by a uniform single-layer adsorption process, but rather a non-uniform multi-layer adsorption process. Additionally, it was observed that the intensity factors (*n_F_*) of biochars ranged from 5.63 to 6.45. This range suggests that the adsorption process is likely to involve multi-layer adsorption on surfaces that are heterogeneous [36]. When the adsorbate is CP, the results are quite different; the correlation coefficients of BCS, BCS-U-T, BCS-K-M, and BCS-H-A were 0.9909, 0.9980, 0.9972, and 0.9969 while the Langmuir model was used, which were all higher than those of the Freundlich model (0.9896 for BCS, 0.9663 for BCS-U-T, 0.9607 for BCS-K-M, and 0.9407 for BCS-H-A). This may be due to the different structures and molecular weights (480.90 for TH and 323.13 for CP) of the adsorbents being adsorbed, and the results show that the adsorption process produces different effects when faced with different adsorbents even if the adsorbents are of the same type.

**Figure 7:**
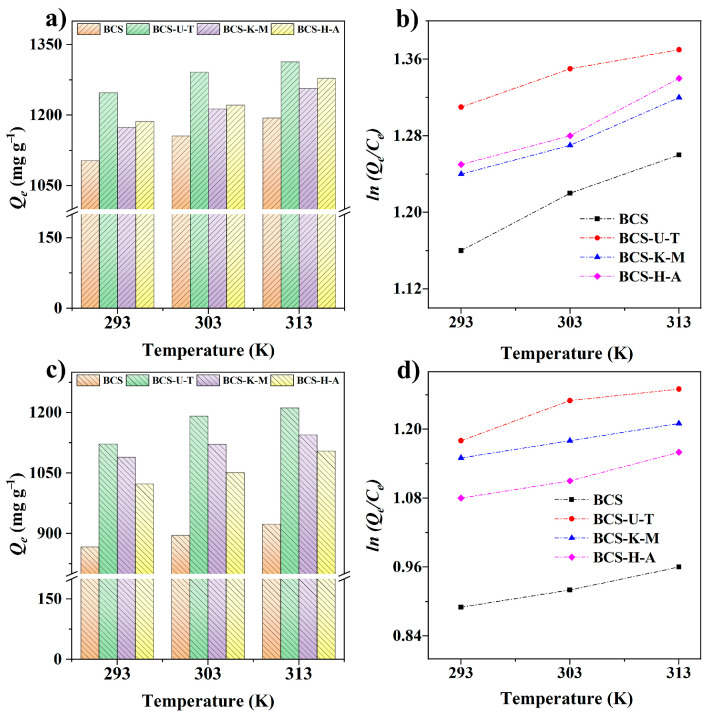
Effects of different temperatures on the adsorption capacities of biochars and the plot of ln(*Q_e_*/*C_e_*) versus T for adsorption of TH (**a**,**b**) and CP (**c**,**d**) (dose of biochars: 10 mg; *V*: 100 mL; *C*_0_: 400 mg/L; pH: 7.0; shaker speed: 150 RPM).

The calculation of thermodynamic parameters is one of the important indicators to explore the adsorption process as affected by temperature. We investigated the impact of varying temperatures (293, 303, and 313 K) on the adsorption process of antibiotics (TH and CP) by biochars (Figure 7). The adsorption capabilities of biochars for TH and CP considerably increased with the temperature rise from 293 K to 303 K, and the trend remained the same with further increases in temperature, indicating that higher temperatures are conducive to promoting the adsorption process. The thermodynamic model was utilized to assess the adsorption data in order to further understand how temperature affects adsorption (Table S5, Supplementary Materials). Δ*G* is used to test the spontaneity of the process under constant-temperature and -pressure conditions [37]. When the Δ*G* value is negative, the reaction process can proceed spontaneously, while when the Δ*G* value is positive, the reaction process tends to proceed spontaneously in the opposite direction. All Δ*G* values were less than zero, demonstrating that adsorption processes occur naturally independent of biochar type (BCS, BCS-U-T, BCS-K-M, and BCS-H-A) or antibiotic (TH or CP). Δ*H* represents the amount of heat absorbed or released during a chemical reaction [38]. The thermodynamic enthalpies Δ*H* of TH adsorption ranged from 18.86 to 21.84 kJ mol^−1^, and those of CP adsorption range from 16.64 to 21.53 kJ mol^−1^, confirming the adsorption process’s endothermic features. Furthermore, the Δ*S* values were all positive (BCS: 3.52 and 2.71 Jmol^−1^ K^−1^ for TH and CP; BCS-U-T: 2.34 and 3.43 Jmol^−1^ K^−1^ for TH and CP; BCS-K-M: 3.05 and 2.19 Jmol^−1^ K^−1^ for TH and CP; BCS-H-A: 3.36 and 3.36 Jmol^−1^ K^−1^ for TH and CP), indicating that the disorder at the interface of the two phases increases with the increase in temperature during the reaction [39].

**Figure 8:**
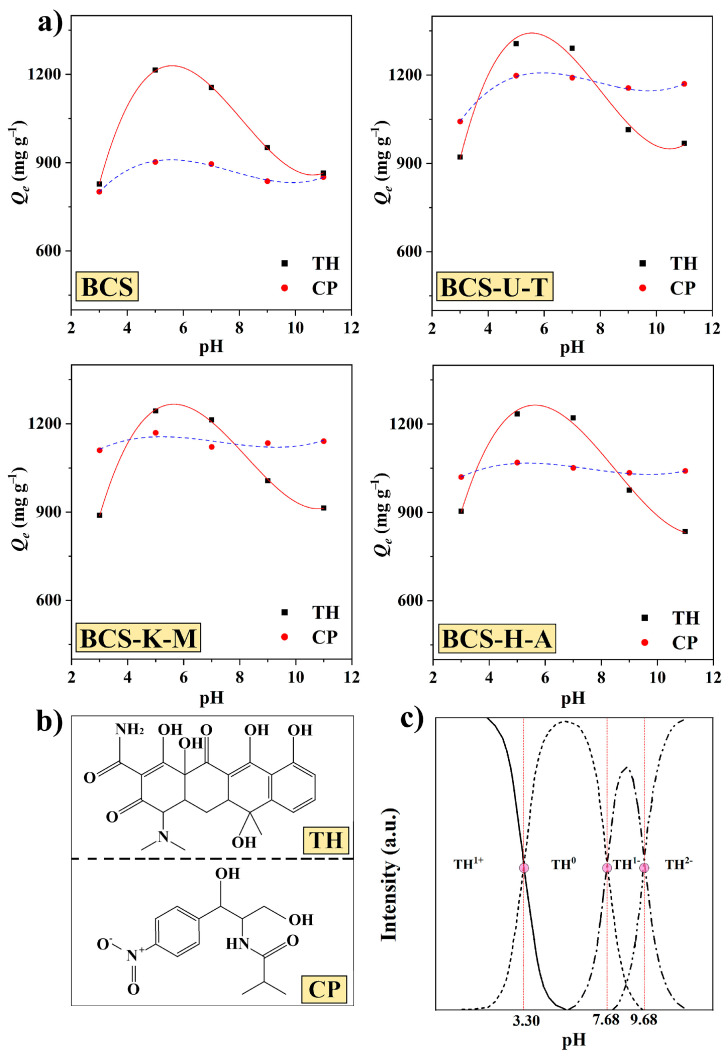
(**a**) Effect of initial pH of solutions on the adsorption capacities of BCS, BCS-U-T, BCS-K-M, and BCS-H-A to TH and CP. (**b**) The chemical structure of TH and CP. (**c**) The existing form of TH at different pH values (dose of biochars: 10 mg; *V*: 100 mL; *C_0_*: 400 mg/L; temperature: 303 K; shaker speed: 150 RPM).

One of the main aspects impacting the adsorption process is the pH value of the solution environment, which frequently impacts the adsorption process by modifying the surface characteristics of the adsorbent and the chemical properties of the adsorbate [40]. We investigated the influence of the initial pH of the solution on antibiotic adsorption by biochars (Figure 8), and it can be noted that with rising initial pH of the solution, TH adsorption displayed a pattern of increasing and then decreasing. Meanwhile, the impact of this technique on CP was minimal. The clear effect of pH on the adsorption of TH by biochars may be because it not only changes the surface electrochemical characteristics of biochars but also has a large influence on the molecular properties of TH. In general, distinct types of TH occur at different pH levels. When the pH is less than 3.30, TH^1+^ dominates and biochars have a positive zeta potential (pH_pzc_ = 4.97 for BCS, pH_pzc_ = 3.34 for BCS-U-T, pH_pzc_ = 4.32 for BCS-K-M, and pH_pzc_ = 4.73 for BCS-H-A). At this point, mutually repellent electrostatic attraction prevents adsorption. TH occurs as TH^0^ when the pH is between 3.30 and 7.68. When the pH value is between 7.68 and 9.68, TH occurs in the form of TH^1−^. When the pH exceeds 9.68, however, TH is dominated by TH^2−^ [41]. The pK_a_ of CP is 5.50 [42], and when the solution pH is close to this value, CP exists in a neutral form (i.e., non-ionized fraction), which explains why the maximum adsorption capacity of biochar for CP occurs at pH values of 4–6.

### 2.4. Adsorption Experiments in Binary System

TH and CP are both broad-spectrum antibiotics, and their mechanism of action is to bind to bacterial ribosomes (TH binds to the 30s subunit of ribosomes and CP binds to the 50s subunit of ribosomes). For some diseases caused by special bacterial infections, doctors will use the two in combination to enhance the therapeutic effect; therefore, it is likely that both antibiotics are present in water. Details of the methodology for the study of binary adsorption systems are given in Supplementary Material S4. The adsorption properties of biochars in the presence of both adsorbents were investigated using the solution equal-volume system (Figure S4). The sum of the capacities of the samples for CP and TH in a binary adsorption system was less than the that of TH in a single system due to the inhibition of adsorption site competition [43,44]. The total adsorption capacity in the binary system is higher than the CP adsorbed in the single system. It can be speculated that the molecular structures of CP and TH contain unsaturated functional groups and hydroxyl, which implies the possibility of hydrogen bonding between antibiotic molecules or between antibiotics and biochar, thus reducing the decrease in adsorption capacity due to inhibition of adsorption site competition.

### 2.5. Reusability Results

The reusability of biochar is the most important index with which to evaluate its practical performance, so we studied the biochars by cycle testing; the details of the methodology for the cycle tests are given in Supplementary Material S4 and the results are shown in Figure S5. The removal rates exhibited a progressive decline as the number of cycles rose. One aspect to consider is that the antibiotics adsorbed onto biochars undergo re-carbonization, resulting in the formation of by-products on the biochar surface. This, in turn, leads to a decrease in the quantity of adsorption sites available for usage. As the regeneration cycles progress, the structure and physicochemical properties of biochar become more fragile, and part of the pore structure may be destroyed during regeneration, which will reduce the performance of biochars. The removal rates of antibiotics by BCS, BCS-U-T, BCS-K-M, and BCS-H-A remained above 80% (after five cycles). This suggests that the biochars exhibited favorable stability and regeneration capabilities in the context of antibiotic removal from water. Remarkably, following five iterations of experimentation, it was observed that the elimination efficiency of antibiotics (whether TH or CP) from straw biochar that underwent mycelium doping exhibited a somewhat superior performance compared to BCS. It can be speculated that mycelium modification improved the mechanical strength and stability of lignocellulosic biochar. Besides the ability to regenerate, the evaluation of adsorbents’ actual performance also relies on their adsorption capacity, which is a crucial characteristic. Hence, a comparison was conducted to assess the adsorption capabilities of BCS, BCS-U-T, BCS-K-M, and BCS-H-A in relation to other adsorbents, including additional charcoal adsorbents (Table S6, Supplementary Materials). The analysis of the adsorption capacity of biochar, as prepared in this work, reveals that it is not characterized by low levels. This finding further underscores the considerable potential and promising prospects of biochar in the context of water antibiotic treatment.

### 2.6. Possible Mechanisms in Adsorption Processes

**Figure 9:**
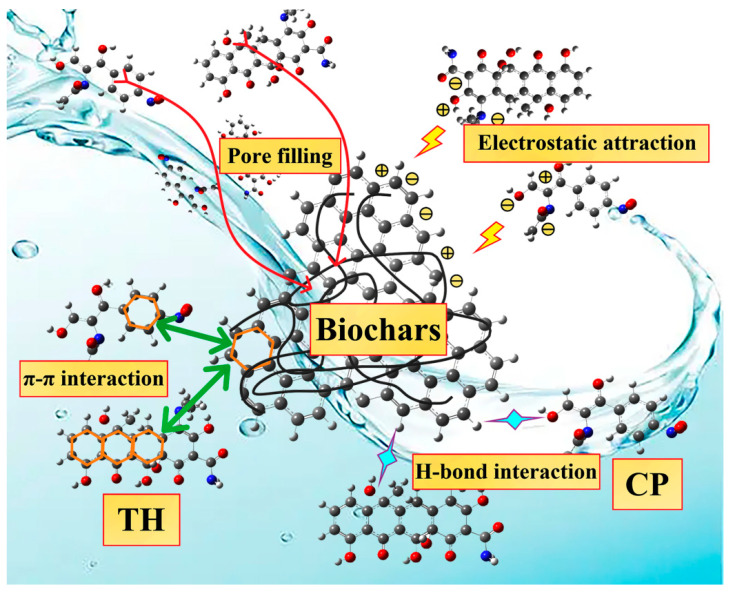
Probable mechanisms of biochars for removal of antibiotics (TH and CP).

In this work, biochars had a large specific surface area and high total pore volume, which facilitated the adsorption of TH and CP by providing numerous adsorption sites. The pore-filling mechanism is considered to be crucial in adsorption. A large number of unsaturated carbon-containing and oxygen-containing functional groups on the surface of the sample, such as C=O, -OH, etc., can form hydrogen bonds with the pollutant molecules, thus enhancing the adsorption and potentially augmenting the adsorption capacity. In addition, the aromatic rings in biochar have the potential to form π–π interactions with the aromatic rings present in antibiotic compounds. Surface-charged biochar can form electrostatic attraction with pollutant molecules of opposite charge, enhancing adsorption. It can be deduced that the exceptional antibiotic removal abilities of BCS, BCS-U-T, BCS-K-M, and BCS-H-A in water are due to the combined effect of the multiple adsorption mechanisms described above (Figure 9).

## 3. Materials and Methods

### 3.1. Materials and Reagents

The corn straw (CS) used in this study was obtained from the Black Soil Experimental Area of Jilin Agricultural University in 2022, and the variety was JINONGYU-1801 (planted under the following growing conditions: N: 150 kg/hm^2^; P_2_O_5_: 70 kg/hm^2^; K_2_O: 70 kg/hm^2^), and the obtained straw leaf to stem ratio was 1:3.5. Subsequently, the straw was washed, dried, pulverized through a 20-mesh sieve, and set aside. The experiment was carried out in 2023 at the Key Laboratory of Straw Comprehensive Utilization and Black Soil Conservation, Ministry of Education.

*Aspergillus niger* (*A. niger*), *Trichoderma reesei* (*T. reesei*), and *Myrothecium verrucaria* (*M. verrucaria*) were provided by the Key Laboratory of Straw Comprehensive Utilization and Black Soil Conservation, Ministry of Education. Chloramphenicol (CP, CAS: 56–75-7) and tetracycline hydrochloride (TH, CAS: 64–75-5) were used as adsorbates for the experiments and provided by Aladdin Chemicals (Shanghai) Co. Other materials and reagents used in the experiments are detailed in Supplementary Material S1. It is worth noting that the reagents used were analytically pure and did not require additional purification.

### 3.2. Preparation Methods

Preparation of treated CS: 10.0 g of corn straw (CS) was introduced into a flask containing a 2 wt% KOH solution (solid–liquid ratio 1:20) and subjected to treatment at 120 °C for 60 min. Another portion of 10.0 g of CS was placed in a flask containing a 2 wt% HCl solution (solid–liquid ratio 1:20) and treated at 110 °C for 60 min. Additionally, 10.0 g of CS was added to a flask containing an 8 wt% KOH and 12 wt% urea solution (solid–liquid ratio 1:20) and treated at 80 °C for 60 min. Following the completion of each reaction, the resulting suspension underwent a sieving process, leading to the collection of the CS residue. The filtered residues were dried to a consistent weight and rinsed with deionized water until reaching a neutral pH. The differently treated CS samples were labeled as CS-K, CS-H, and CS-U, respectively, representing the treating agents of KOH, HCl, and urea/KOH.

Preparation of fungal spore suspensions: A potato glucose liquid culture medium was formulated, consisting of 200.0 g L^−1^ potato extract and 20.0 g L^−1^ D-(+)-glucose monohydrate. This medium was sterilized at 115 °C for 30 min, subsequently cooled, and inoculated with the desired fungal strains. Fungal spore suspensions were generated by incubating the inoculated medium on a shaking incubator set at 150 RPM and 30.0 °C for a duration of 5 days.

Preparation of inorganic nutrient solution [3]: In total, 2.0 g L^−1^ of (NH_4_)_2_SO_4_, 2.0 g L^−1^ of KH_2_PO_4_, 0.3 g L^−1^ of MgSO_4_, 0.3 g L^−1^ of CaCl_2_, 0.5 g L^−1^ of NaCl, 0.005 g L^−1^ of FeSO_4_, 0.016 g L^−1^ of MnSO_4_, and 0.017 g L^−1^ of ZnCl_2_ were thoroughly mixed.

Preparation of straw solid medium: 10.0 g of pretreated CS was stirred thoroughly with 30.0 mL of inorganic nutrient solution, sterilized at 115 °C for 30 min, and cooled to room temperature.

Preparation of mycelium-doped straw [21]: In total, 200 uL of suspension containing *Aspergillus niger* (*A. niger*), *Trichoderma reesei* (*T. reesei*), and *Myrothecium verrucaria* (*M. verrucaria*) spores was added into straw solid medium, sealed, stirred well, and incubated in the incubator at 30 °C for 14 days. Then, the mycelium-doped straw samples were dried at 80 °C for 12 h. The different mycelium-doped CSs were denoted as CS-H-A, CS-U-T, and CS-K-M, respectively, representing CS-H, CS-U, and CS-K doped by *A. niger*, *T. reesei*, and *M. verrucaria*.

Preparation of biochar: The preparation of biochar was divided into two steps. CS-H-A, CS-U-T, and CS-K-M were pyrolyzed at 600 °C for 60 min with the heating rate of 10 °C min−1 under the protection of nitrogen flow. After cooling down to room temperature, carbonized samples (CCS-H-A, CCS-U-T, and CCS-K-M) were activated by KOH at a ratio of 1:4 (*w*:*w*) at 700 °C for 60 min. The mixtures were washed with 0.1 M HCl and deionized water to obtain biochar. The different biochars were denoted as BCS-H-A, BCS-U-T, and BCS-K-M, respectively, obtained from CCS-H-A, CCS-U-T, and CCS-K-M. Characterization test details for all samples are provided in Supplementary Material S2.

## 4. Conclusions

In this study, chemical pretreatment combined with microbial solid fermentation was used to treat corn straw, and then the straw biomass modified by fungal mycelia was prepared into biochars (BCS-U-T, BCS-K-M, and BCS-H-A) by the carbonation activation method. The biochars exhibited enhanced specific surface area, total pore volume, and physical and chemical characteristics as compared to the original straw biochar (BCS). The removal studies conducted using TH and CP as adsorbates showed exceptional treatment capabilities. Furthermore, it is noteworthy that even after undergoing five regeneration cycles, the removal rates of biochars remain above 80%. This observation underscores the significant potential of biochar in many practical applications. This study not only produced a batch of biochar with enhanced performance for water antibiotic treatment, but also demonstrated the viability of fungal mycelium modification to further enhance biochar performance. Moreover, the study presented innovative ideas and methods for the holistic utilization of straw and other economically significant crops.

## Data Availability

Data contained within the article.

## Supplementary Materials

The following supporting information can be downloaded at https://www.mdpi.com/article/10.3390/ijms252111387/s1 [45,46,47,48,49,50,51,52,53,54].
