# Supplementary material for: Mycelium-Doped Straw Biochars for Antibiotic Control
Source: Int J Mol Sci. 2024 Oct 23;25(21):11387. doi: 10.3390/ijms252111387 (PMC11546586; doi:10.3390/ijms252111387)
Supplement: Supplementary file 1 [file ijms-25-11387-s001.zip › ijms-3260125-supplementary.pdf]

Mycelium-Doped Straw Biochars for Antibiotic Control

Bolun Zhang [1,2], Ruqi Li [1,2], Huiji Zhang [1,2], Ye Han [1,2], Yunzhe Jia [1,2], Siji Chen [1,2,*] and Xiaoxiao Yu [1,2,*]

[1] College of Life Sciences, Jilin Agricultural University, Changchun 130118, China; aa921526347@163.com (B.Z.); 14752267617@163.com (R.L.); zhanghuiji2001@163.com (H.Z.); hy2591203582@163.com (Y.H.); j18088671670@163.com (Y.J.)

[2] Key Laboratory of Straw Comprehensive Utilization and Black Soil Conservation, Ministry of Education, Jilin Agricultural University, Changchun 130118, China

[*] Correspondence: sijichen@jlau.edu.cn (S.C.); xiaoxiaoy@jlau.edu.cn (X.Y.)

S1 Materials and reagents

D-(+)-Glucose monohydrate (CAS: 14431-43-7), ammonium sulfate ($(NH_4)_2SO_4$, CAS: 7783-20-2), potassium dihydrogen phosphate (KH_2PO_4, CAS: 7778-77-0), magnesium sulfate ($MgSO_4$, CAS: 7487-88-9), calcium chloride ($CaCl_2$, CAS: 10043-52-4), sodium chloride (NaCl, CAS: 7647-14-5), iron sulfate heptahydrate ($FeSO_4 \cdot 7H_2O$, CAS: 7782-63-0), manganese sulfate monohydrate ($MnSO_4 \cdot H_2O$, CAS: 10034-96-5), zinc chloride ($ZnCl_2$, CAS: 7646-85-7), urea (CAS: 57-13-6), sodium hydroxide (NaOH, CAS: 1310-73-2), potassium hydroxide (KOH, CAS: 1310-58-3), and hydrochloric acid (HCl, CAS: 7647-01-0) were supplied by Aladdin Chemical (Shanghai) Co. Ltd. (Shanghai, China). Potatoes were bought from a local supermarket.

S2 Characterization methods

Scanning electron microscopy (SEM, Zeiss Gemini 500, Germany) was used to examine the morphology of materials. FT-IR spectrometer was used to characterize the surface functional groups of materials between 400 and 4000 cm^{-1} at a resolution of 1 cm^{-1} (FT-IR, Thermo Fisher Nicolet iS50, USA). X-ray diffraction patterns of the powders were observed by an X-ray diffractometer with a filtered Cu-Ka X-ray

source (XRD, Bruker D8 Advance, Germany). Raman spectra of the samples were obtained using a model Renishaw 2000 Raman spectrometer at 514 nm to investigate the presence of defects in the biochar materials. X-ray photoelectron spectroscopy was used to test the electronic binding energy of the samples (XPS, Thermo Escalab 250Xi^{+}, USA). N_2 adsorption-desorption isotherms were used to obtain the porosity of the samples at 77 K (N_2 adsorption-desorption isotherms, Micromeritics ASAP 2020 Plus Version 2.00, USA). The zeta potential instrument was used to characterize the surface charge of samples (Zeta potential, Zetasizer Nano ZS90, UK). Composition analysis of pretreated corn straw: the cellulose, lignin, and hemicellulose contents were analyzed based on the method described by the National Renewable Energy Laboratory (NREL).

S3 Adsorption experiments methodology

BCS, BCS-H-A, BCS-U-T, and BCS-K-M were added to a flask containing different antibiotics solutions, including TH or CP. The flask was positioned within constant temperature shaker (150 rpm, dark condition). The effects of initial concentrations (100-500 mg L^{-1}), adsorption time (0-150 min), adsorption temperature (293-313 K), and pH (adjusted to different pH values by 0.1 M NaOH or HCl) were investigated for the removal of antibiotics. After the equilibrium adsorptions were reached, the concentrations of the solutions were determined by a spectrophotometer (Agilent Cary-300 UV-vis). The adsorption capacity (Q_e, mg g^{-1}) and the removal rate (R%) were calculated by Eqs. (1-2):

$$Q_e = \frac{(C_0 - C_e) \times V}{m} \tag{1}$$

$$R\% = \frac{C_0 - C_e}{C_0} \times 100\% \tag{2}$$

where C_e (mg L^{-1}), C_0 (mg L^{-1}), m (g), and V (L) denote the equilibrium concentrations, initial concentrations, quality of biochar, and solutions volume, respectively.

The adsorption kinetic experiments and the adsorption isotherm experiments were performed at 303 K. Pseudo-first-order (PFO, Ep. 3), pseudo-second-order (PSO, Ep.

4), and intra-particle diffusion (IPD, Ep. 5) models were used to analyze the adsorption kinetics of antibiotics. The expressions for models were presented as follow:

$$Q_t = Q_e(1 - \frac{1}{e^{k_1 t}}) \tag{3}$$

$$Q_t = \frac{k_2 Q_e^2 t}{1 + k_2 Q_e t} \tag{4}$$

$$Q_t = k_3 t^{0.5} + C \tag{5}$$

where Q_t (mg g^{-1}), k_1, k_2, k_3, and C denote the adsorption capabilities of samples at various time intervals, the rate constants of the PFO, PSO, and IPD kinetic models, and the thickness of the boundary layer constant, respectively.

Langmuir isotherm model (Eq. 6), Freundlich isotherm model (Eq. 7), and Temkin isotherm model (Eq. 8) were used to analyze the adsorption isotherms of antibiotics. The expressions for models were presented as follow:

$$Q_e = \frac{Q_m K_L C_e}{1 + K_L C_e} \tag{6}$$

$$Q_e = K_F C_e^{1/n_F} \tag{7}$$

$$Q_e = b(\ln K_T + \ln C_e) \tag{8}$$

where Q_m (mg g^{-1}) is the maximum adsorption capacities of adsorbents calculated by the model, K_L, K_F, and b denote the Langmuir, Freundlich, and Temkin adsorption isotherm constants, n_F and b denote Freundlich linearity index and Temkin equilibrium constant.

The adsorption thermodynamic experiments were performed with the initial concentration of 200 mg L^{-1}. The adsorption processes were analyzed by calculating the thermodynamic parameters. The expressions for models were presented as follow:

$$\ln(\frac{Q_e}{C_e}) = \frac{\Delta S}{R} - \frac{\Delta H}{RT} \tag{9}$$

$$\Delta H = \Delta G + T\Delta S \tag{10}$$

where ΔS, ΔG, ΔH, and R denote standard entropy, free gibbs energy, enthalpy, and gas constant (8.314 J/K·mol).

S4 Binary adsorption system study and cycle test

To investigate the effect of different antibiotics on the adsorption capacities of adsorbents, the different antibiotics solution with different combinations, including Tetracycline hydrochloride (TH), Chloramphenicol (CP), and TH-CP (equal concentration of TH mixed with CP), with different initial total concentrations of 100, 200, and 400 mg L^{-1} were prepared (such as the initial total concentration is 200 mg L^{-1}, that is, 200 mg L^{-1} of TH + 0 mg L^{-1} of CP, 100 mg L^{-1} of TH + 100 mg L^{-1} of CP, and 0 mg L^{-1} of TH + 200 mg L^{-1} of CP) and the adsorption experiments were performed at 303 K.

The adsorbents were recycled after being subjected to an adsorption procedure by centrifugation, re-carbonized at 600 °C for 60 min, and reused as fresh adsorbents in subsequent cycles.

Table S1 Changes of main components of CS before and after treatment. Notes: Values of cellulose, hemicellulose and lignin were presented as the relative content.

Samples	Cellulose (%)	Hemicellulose (%)	Lignin (%)
CS	38.15±2.06	21.52±1.47	22.26±2.14
CS-H	55.43±1.42	6.50±0.68	19.77±1.15
CS-K	49.43±1.58	20.44±1.58	10.39±1.00
CS-U	15.88±2.54	18.42±3.01	29.71±2.30

Table S2 Textural data of samples obtained on the basis of N_2 adsorption-desorption.

Samples	S_{BET} (m^2 g^{-1})	V_{total} (cm^3 g^{-1})	P_m (nm)
BCS	2429	1.30	2.14
BCS-U-T	2604	1.42	2.18
BCS-K-M	2790	1.51	2.16
BCS-H-A	2715	1.54	2.26

Note: S_{BET}, the BET surface area, in $m^2\ g^{-1}$; Vtotal, the total pore volume, in $cm^3\ g^{-1}$; Pm, the mean pore size, in nm.

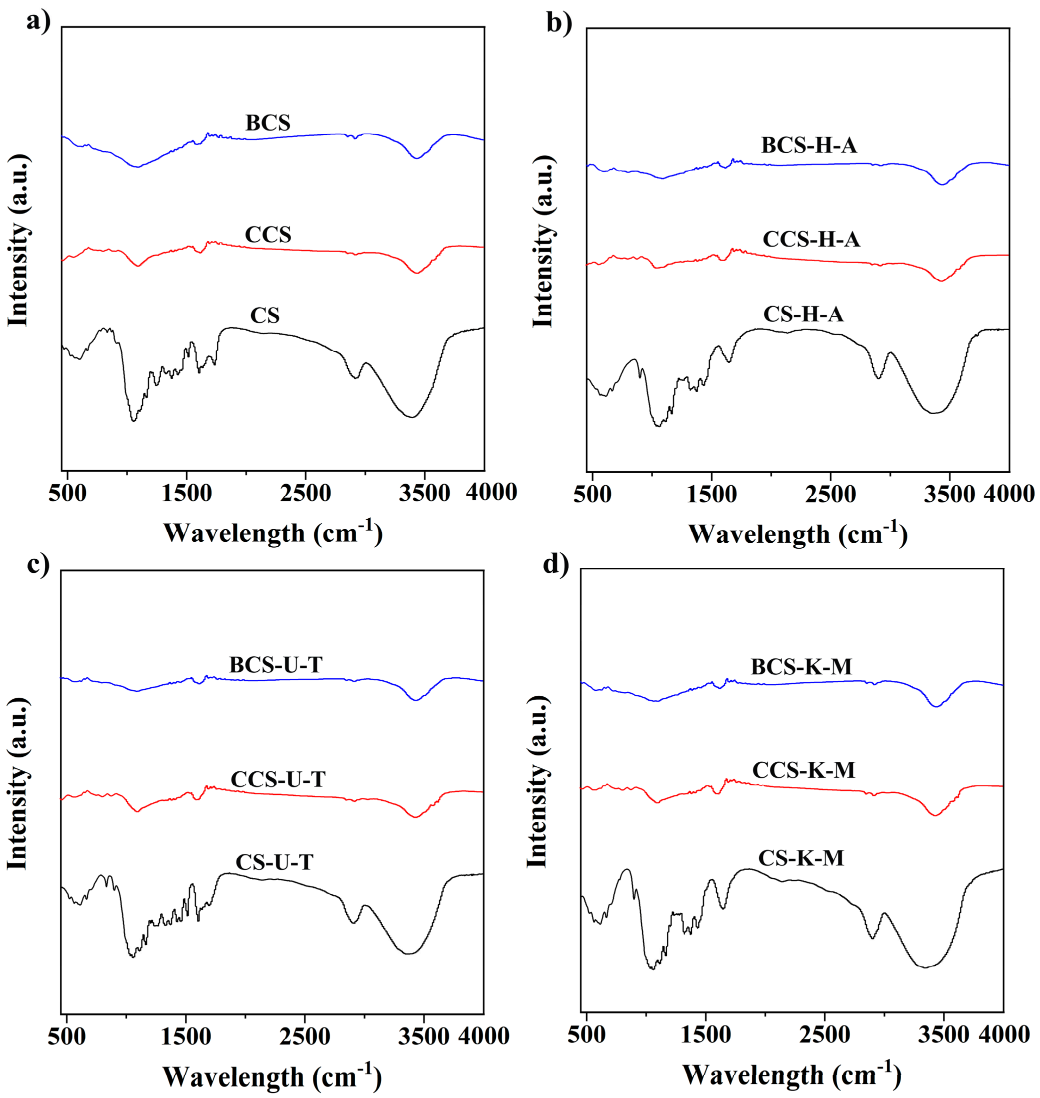

Fig. S1 FT-IR tests of (a) CS, CCS, and BCS, (b) CS-H-A, CCS-H-A, and BCS-H-A, (c) CS-U-T, CCS-U-T, and BCS-U-T, (d) CS-K-M, CCS-K-M, and BCS-K-M.

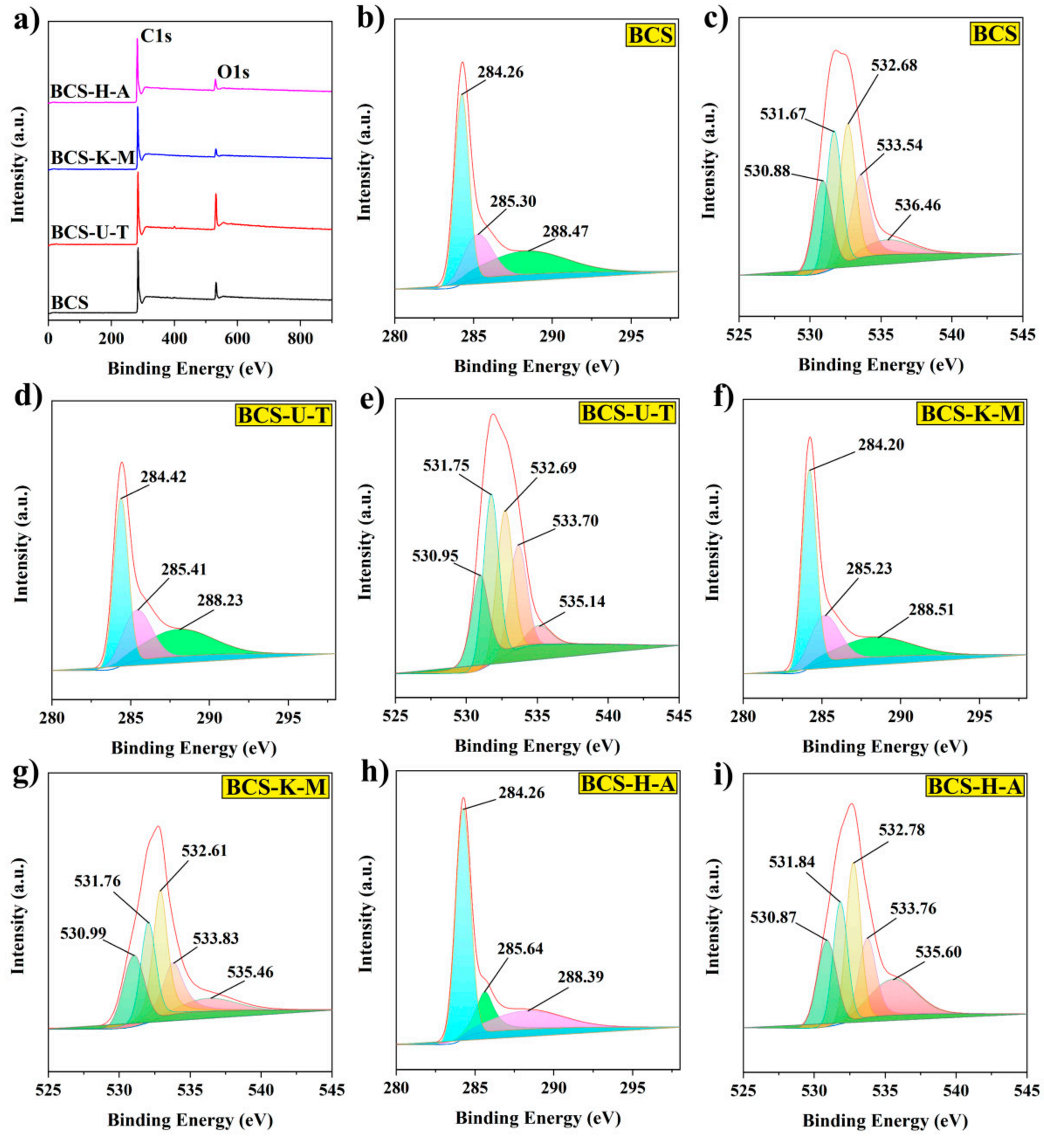

Fig. S2 (a) XPS tests of BCS, BCS-U-T, BCS-H-A, and BCS-K-M. The (b) C1s and (c) O1s of BCS. The (d) C1s and (e) O1s of BCS-U-T. The (f) C1s and (g) O1s of BCS-K-M. The (h) C1s and (i) O1s of BCS-H-A.

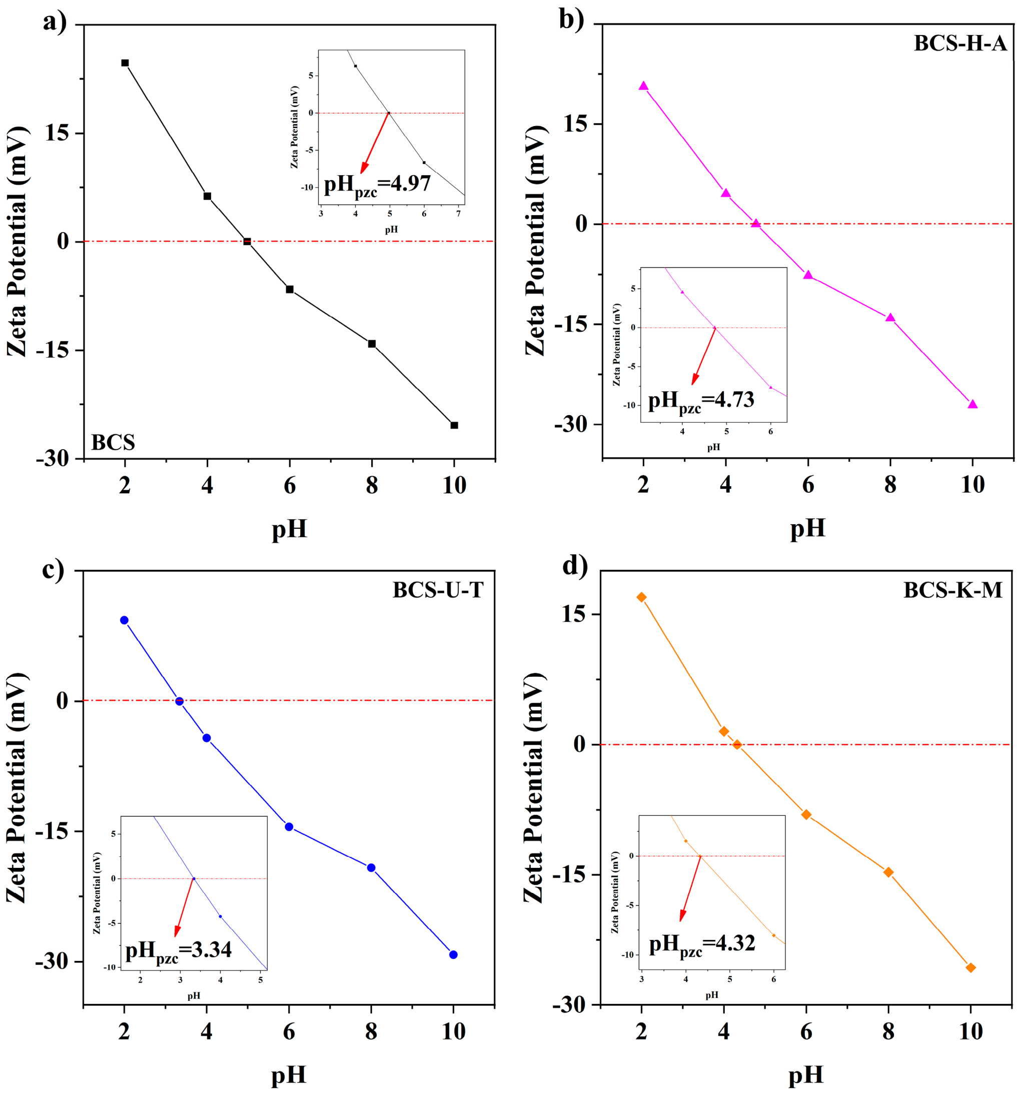

Fig. S3 Zeta potential of (a) BCS, (b) BCS-H-A, (c) BCS-U-T, and (d) BCS-K-M.

Table S3 Fitting parameters of adsorption kinetic models for TH and CP at 303 K.

Adsorbates	Adsorbents	Models	Parameters	C_0 (mg L^{-1})		
				100	200	300
TH	BCS	PFO	Q_e (mg g^{-1})	864.77	985.65	1080.77
			k_1 (min^{-1})	0.0086	0.0068	0.0062
			$Q_{e.cat}$ (mg g^{-1})	827.88	942.28	1055.60
			R^2	0.9849	0.9802	0.9869
		PSO	k_2 (g mg^{-1} min^{-1})	0.0008	0.0012	0.0019
			$Q_{e.cat}$ (mg g^{-1})	867.14	986.19	1081.96
			R^2	0.9971	0.9996	0.9996

	IPD	k_3 (mg g^{-1} min$^{-0.5}$)	15.51	11.50	8.39
		C	697.05	862.06	986.60
		R^2	0.8834	0.7952	0.8607
BCS-U-T	PFO	Q_e (mg g^{-1})	939.49	1110.44	1239.68
		k_1 (min^{-1})	0.008	0.0089	0.4438
		$Q_{e.cat}$ (mg g^{-1})	882.01	1051.97	1187.48
		R^2	0.9676	0.9770	0.9757
	PSO	k_2 (g mg^{-1} min^{-1})	0.0006	0.0006	0.0008
		$Q_{e.cat}$ (mg g^{-1})	944.43	1111.39	1243.73
		R^2	0.9913	0.9947	0.9993
	IPD	k_3 (mg g^{-1} min$^{-0.5}$)	22.88	23.87	15.79
		C	688.83	850.40	1077.40
		R^2	0.9331	0.9135	0.7652
BCS-K-M	PFO	Q_e (mg g^{-1})	908.79	1046.32	1130.98
		k_1 (min^{-1})	0.0101	0.0053	0.0063
		$Q_{e.cat}$ (mg g^{-1})	848.52	982.70	1080.36
		R^2	0.9747	0.9748	0.9774
	PSO	k_2 (g mg^{-1} min^{-1})	0.0007	0.0004	0.0004
		$Q_{e.cat}$ (mg g^{-1})	918.14	1052.22	1132.96
		R^2	0.9918	0.9954	0.9970
	IPD	k_3 (mg g^{-1} min$^{-0.5}$)	19.20	26.83	27.88
		C	688.95	750.13	834.95
		R^2	0.9556	0.8863	0.8164
BCS-H-A	PFO	Q_e (mg g^{-1})	896.34	1032.23	1105.02
		k_1 (min^{-1})	0.0089	0.0109	0.0167
		$Q_{e.cat}$ (mg g^{-1})	859.17	1006.33	1129.74
		R^2	0.9856	0.9856	0.9873
	PSO	k_2 (g mg^{-1} min^{-1})	0.0009	0.0012	0.0019
		$Q_{e.cat}$ (mg g^{-1})	898.46	1034.64	1109.13

			R^2	0.9996	0.9973	0.9995
			k_3 (mg g^{-1} min$^{-0.5}$)	15.51	11.50	8.39
		IPD	C	728.62	908.66	1060.85
			R^2	0.8652	0.8001	0.8607
			Q_e (mg g^{-1})	671.07	792.41	858.81
		PFO	k_1 (min^{-1})	0.0065	0.0057	0.0060
			$Q_{e.cat}$ (mg g^{-1})	632.45	763.59	827.95
			R^2	0.9671	0.9884	0.9881
			k_2 (g mg^{-1} min^{-1})	0.0007	0.0005	0.0005
	BCS	PSO	$Q_{e.cat}$ (mg g^{-1})	675.50	802.49	867.87
			R^2	0.9929	0.9998	0.9998
			k_3 (mg g^{-1} min$^{-0.5}$)	17.63	20.77	21.20
		IPD	C	479.90	575.18	637.13
			R^2	0.8794	0.7407	0.7496
			Q_e (mg g^{-1})	807.89	1040.78	1128.63
		PFO	k_1 (min^{-1})	0.0046	0.0089	0.0091
CP			$Q_{e.cat}$ (mg g^{-1})	735.46	1010.03	1090.52
			R^2	0.8959	0.9881	0.9889
			k_2 (g mg^{-1} min^{-1})	0.0005	0.0008	0.0006
	BCS-U-T	PSO	$Q_{e.cat}$ (mg g^{-1})	817.71	1041.04	1130.10
			R^2	0.9998	0.9992	0.9999
			k_3 (mg g^{-1} min$^{-0.5}$)	20.77	15.83	20.52
		IPD	C	590.66	873.23	909.16
			R^2	0.7416	0.7626	0.7349
			Q_e (mg g^{-1})	768.67	997.54	1065.72
		PFO	k_1 (min^{-1})	0.0081	0.0082	0.0076
	BCS-K-M		$Q_{e.cat}$ (mg g^{-1})	740.93	956.50	1030.00
			R^2	0.9867	0.9861	0.9875
		PSO	k_2 (g mg^{-1} min^{-1})	0.0007	0.0006	0.0006

		$Q_{e.cat}$ (mg g^{-1})	771.04	998.39	1070.03
		R^2	0.9988	0.9990	0.9998
	IPD	k_3 (mg g^{-1} min$^{-0.5}$)	15.83	21.18	20.94
		C	601.12	771.01	844.62
		R^2	0.7626	0.8061	0.7467
BCS-H-A	PFO	Q_e (mg g^{-1})	758.27	964.53	1019.80
		k_1 (min^{-1})	0.0082	0.0074	0.0077
		$Q_{e.cat}$ (mg g^{-1})	717.79	932.69	986.36
		R^2	0.9723	0.9866	0.9864
	PSO	k_2 (g mg^{-1} min^{-1})	0.0007	0.0005	0.0006
		$Q_{e.cat}$ (mg g^{-1})	761.09	972.19	1026.18
		R^2	0.9939	0.9998	0.9998
	IPD	k_3 (mg g^{-1} min$^{-0.5}$)	17.63	20.77	21.20
		C	567.11	747.31	798.12
		R^2	0.8794	0.7406	0.7497

Table S4 Fitting parameters of adsorption isotherm models for TH and CP at 303 K.

Adsorbates	Adsorbents	Types	Parameters	
TH	BCS	Langmuir	Q_m (mg g^{-1})	1236.16
			K_L (L mg^{-1})	0.0363
			R^2	0.8942
		Freundlich	K_F (mg g^{-1}(L mg^{-1})$^{1/n}$)	430.57
			n_F	5.63
			R^2	0.9908
	BCS-U-T	Langmuir	Q_m (mg g^{-1})	1394.89
			K_L (L mg^{-1})	0.0356
			R^2	0.9490
		Freundlich	K_F (mg g^{-1}(L mg^{-1})$^{1/n}$)	473.97
			n_F	5.79

			R^2	0.9950
	BCS-K-M	Langmuir	Q_m (mg g^{-1})	1277.45
			K_L (L mg^{-1})	0.0410
			R^2	0.9223
		Freundlich	K_F (mg g^{-1}(L mg^{-1})$^{1/n}$)	485.96
			n_F	6.45
			R^2	0.9951
	BCS-H-A	Langmuir	Q_m (mg g^{-1})	1296.43
			K_L (L mg^{-1})	0.0367
			R^2	0.9293
		Freundlich	K_F (mg g^{-1}(L mg^{-1})$^{1/n}$)	457.61
			n_F	6.01
			R^2	0.9982
CP	BCS	Langmuir	Q_m (mg g^{-1})	971.15
			K_L (L mg^{-1})	0.0320
			R^2	0.9909
		Freundlich	K_F (mg g^{-1}(L mg^{-1})$^{1/n}$)	340.63
			n_F	6.08
			R^2	0.9896
	BCS-U-T	Langmuir	Q_m (mg g^{-1})	1316.54
			K_L (L mg^{-1})	0.0262
			R^2	0.9980
		Freundlich	K_F (mg g^{-1}(L mg^{-1})$^{1/n}$)	374.69
			n_F	5.06
			R^2	0.9663
	BCS-K-M	Langmuir	Q_m (mg g^{-1})	1246.94
			K_L (L mg^{-1})	0.0260
			R^2	0.9972
		Freundlich	K_F (mg g^{-1}(L mg^{-1})$^{1/n}$)	358.47

		n_F	5.12
		R^2	0.9607
BCS-H-A	Langmuir	Q_m (mg g^{-1})	1156.04
		K_L (L mg^{-1})	0.0311
		R^2	0.9969
	Freundlich	K_F (mg g^{-1}(L mg^{-1})$^{1/n}$)	394.42
		n_F	5.92
		R^2	0.9407

Table S5 Fitting adsorption thermodynamic parameters for TH and CP.

Adsorbents	Adsorbates	T (K)	Δ*G* (kJ mol^{-1})	Δ*H* (kJ mol^{-1})	Δ*S* (J mol^{-1} K^{-1})
BCS	TH	293	-2.83	21.69	3.52
		303	-3.07		
		313	-3.27		
	CP	293	-2.16	16.64	2.71
		303	-2.33		
		313	-2.49		
BCS-U-T	TH	293	-3.18	18.86	2.34
		303	-3.40		
		313	-3.56		
	CP	293	-2.88	21.53	3.43
		303	-3.16		
		313	-3.31		
BCS-K-M	TH	293	-3.01	20.69	3.05
		303	-3.21		
		313	-3.42		
	CP	293	-2.80	17.01	2.19
		303	-2.98		
		313	-3.14		
BCS-H-A	TH	293	-3.04	21.84	3.36

	303	-3.23		
	313	-3.48		
	293	-2.62		
CP	303	-2.79	20.43	3.36
	313	-3.03		

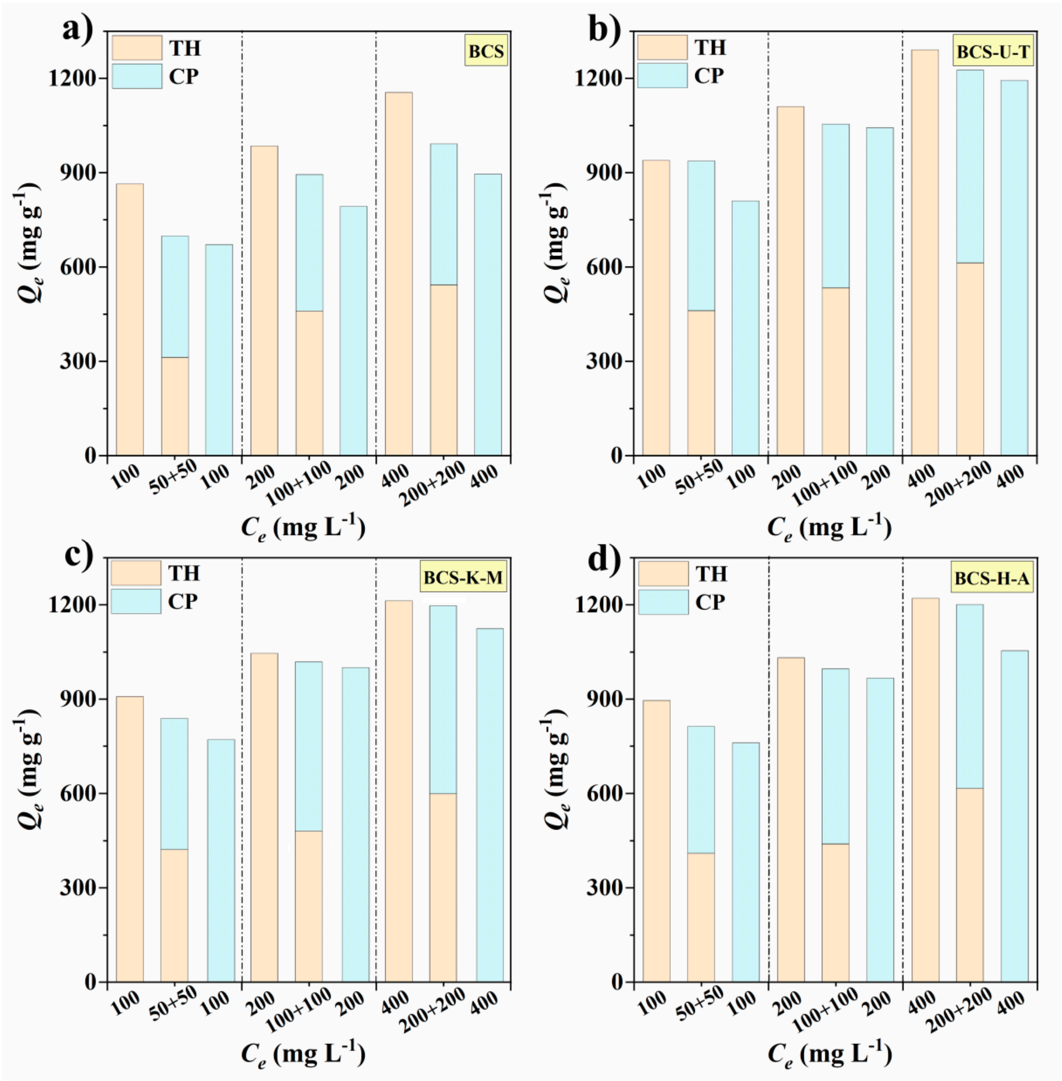

Fig. S4 The adsorption capacities of (a) BCS, (b) BCS-U-T, (c) BCS-K-M, and (d) BCS-H-A to TH and CP in single and binary system at 303 K.

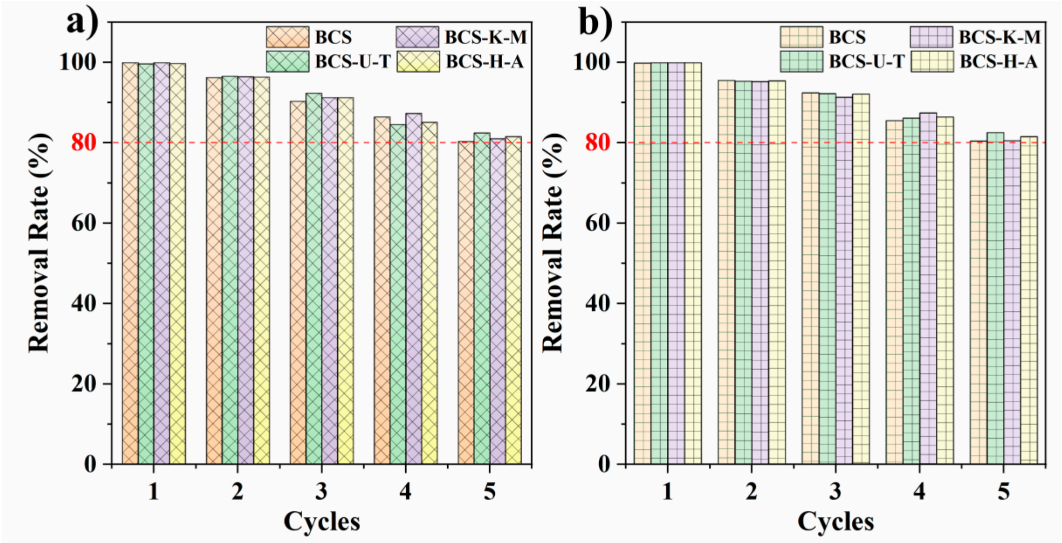

Fig. S5 Reusability of BCS, BCS-U-T, BCS-K-M, and BCS-H-A to (a) TH and (b) CP.

Table S6 Comparison of BCS, BCS-U-T, BCS-K-M, and BCS-H-A to TH and CP with other adsorbents.

Adsorbents	Q_e for TH (mg g^{-1})	Q_e for CP (mg g^{-1})	References
BCS	1080.77	858.81	This work
BCS-K-M	1130.98	1065.72	This work
BCS-H-A	1155.02	1019.80	This work
BCS-U-T	1239.68	1128.63	This work
Biochar-based gel pellets	206.98	-	[S1]
Wheat straw biochar	584.19	-	[S2]
Sunflower seed husk biochar	673.00	-	[S3]
Carbon-Fe_3C/lignin composites	760.36	-	[S4]
Eucommia ulmoides lignin-based biochar	1163.00	-	[S5]
Biochar from lignocellulosic feedstocks	-	11.30	[S6]
Acid-leached halloysites	-	197.20	[S7]
Functionalized biochar	-	200.50	[S8]

Peanut shell biochar	-	423.70	[S9]
Hierarchical porous biochar	-	892.90	[S10]

References

[S1] B. Zhou, Q. Sang, Y. Wang, H. Huang, F. Wang, R. Yang, Y. Zhao, Z. Xiao, C. Zhang, H. Li, Comprehensive understanding of tetracycline hydrochloride adsorption mechanism onto biochar-based gel pellets based on the combination of characterization-based and approximate site energy distribution methods, J. Clean. Prod. 416 (2023) 137909.

[S2] J. Xu, Y. Zhang, B. Li, S. Fan, H. Xu, D. Guan, Improved adsorption properties of tetracycline on KOH/$KMnO_4$ modified biochar derived from wheat straw, Chemosphere. 296 (2022) 133981.

[S3] T. Nguyen, T. Nguyen, W. Chen, C. Chen, X. Bui, A. K. Patel, C. Dong, Hydrothermal and pyrolytic conversion of sunflower seed husk into novel porous biochar for efficient adsorption of tetracycline, Bioresour. Technol. 373 (2023) 128711.

[S4] D. Liu, W. Gu, L. Zhou, J. Lei, L. Wang, J. Zhang, Y. Liu, From biochar to functions: Lignin induced formation of Fe_3C in carbon/Fe composites for efficient adsorption of tetracycline from wastewater, Sep. Purif. Technol. 304 (2023) 122217.

[S5] Y. Chen, J. Liu, Q. Zeng, Z. Liang, X. Ye, Y. Lv, M. Liu, Preparation of *Eucommia ulmoides* lignin-based high-performance biochar containing sulfonic group: Synergistic pyrolysis mechanism and tetracycline hydrochloride adsorption, Bioresour. Technol. 329 (2021) 124856.

[S6] I. Kozyatnyk, P. Oesterle, C. Wurzer, O. Masek, S. Jansson, Removal of contaminants of emerging concern from multicomponent systems using carbon dioxide activated biochar from lignocellulosic feedstocks, Bioresour. Technol. 340 (2021) 125561.

[S7] M. Khelifa, S. Mellouk, G. L. Lecomte-Nana, I. Batonneau-Gener, K. Marouf-Khelifa, A. Khelifa, Methodological approach to the chloramphenicol adsorption by acid-leached halloysites: Preparation, characterization, performance and mechanism, Micropor. Mesopor. Mat. 348 (2023) 112412.

[S8] M. B. Ahmed, J. L. Zhou, H. H. Ngo, W. Guo, M. A. H. Johir, K. Sornalingam, M. S. Rahman, Chloramphenicol interaction with functionalized biochar in water: sorptive mechanism, molecular imprinting effect and repeatable application, Sci. Total Environ. 609 (2017) 885-895.

[S9] J. Yang, G. Ji, Y. Gao, W. Fu, M. Irfan, L. Mu, Y. Zhanga, A. Li, High-yield and high-performance porous biochar produced from pyrolysis of peanut shell with low-dose ammonium polyphosphate for chloramphenicol adsorption, J. Clean. Prod. 264 (2020) 121516.

[S10] G. Wang, X. Yong, L. Luo, S. Yan, J. W. C. Wong, J. Zhou, Structure-performance correlation of high surface area and hierarchical porous biochars as chloramphenicol adsorbents, Sep. Purif. Technol. 296 (2022) 121374.
